# Light Triggered Enhancement of Antibiotic Efficacy in Biofilm Elimination Mediated by Gold-Silver Alloy Nanoparticles

**DOI:** 10.3389/fmicb.2022.841124

**Published:** 2022-02-28

**Authors:** Cinthia Alves-Barroco, Lorenzo Rivas-García, Alexandra R. Fernandes, Pedro Viana Baptista

**Affiliations:** ^1^Applied Molecular Biosciences Unit, Dept. Ciências da Vida, NOVA School of Science and Technology, Costa da Caparica, Portugal; ^2^i4HB, Associate Laboratory–Institute for Health and Bioeconomy, Faculdade de Ciências e Tecnologia, Universidade NOVA de Lisboa, Caparica, Portugal; ^3^Biomedical Research Centre, Institute of Nutrition and Food Technology, Department of Physiology, Faculty of Pharmacy, University of Granada, Granada, Spain

**Keywords:** gold-silver alloy nanoparticles, eradication mature biofilms, biofilm inhibition, biofilms, *Streptococcus*

## Abstract

Bacterial biofilm is a tri-dimensional complex community of cells at different metabolic stages involved in a matrix of self-produced extracellular polymeric substances. Biofilm formation is part of a defense mechanism that allows the bacteria to survive in hostile environments, such as increasing resistance or tolerance to antimicrobial agents, causing persistent infections hard to treat and impair disease eradication. One such example is bovine mastitis associated with *Streptococcus dysgalactiae* subsp. *dysgalactiae* (SDSD), whose worldwide health and economic impact is on the surge. As such, non-conventional nanobased approaches have been proposed as an alternative to tackle biofilm formation and to which pathogenic bacteria fail to adapt. Among these, metallic nanoparticles have gained significant attention, particularly gold and silver nanoparticles, due to their ease of synthesis and impact against microorganism growth. This study provides a proof-of-concept investigation into the use of gold-silver alloy nanoparticles (AuAgNPs) toward eradication of bacterial biofilms. Upon visible light irradiation of AuAgNPs there was considerable disturbance of the biofilms’ matrix. The hindering of structural integrity of the biofilm matrix resulted in an increased permeability for entry of antibiotics, which then cause the eradication of biofilm and inhibit subsequent biofilm formation. Additionally, our results that AuAgNPs inhibited the formation of SDSD biofilms *via* distinct stress pathways that lead to the downregulation of two genes critical for biofilm production, namely, *brpA*-like encoding biofilm regulatory protein and *fbpA* fibronectin-binding protein A. This study provides useful information to assist the development of nanoparticle-based strategies for the active treatment of biofilm-related infections triggered by photoirradiation in the visible.

## Introduction

Biofilms and their associated infections are a significant medical apprehension, which often climax in life-threatening effects (Alves-barroco et al., 2020a). A biofilm is formed by bacteria and is formed by a mass of microbial cells embedded in matrix of extracellular polymeric substances (EPS) produced by the bacteria cluster (Alves-barroco et al., 2020a). These biofilms are extremely infectious and seem to be present throughout the environment, and wherever the microbes adsorb on and/or proliferate toward colonization (Marks et al., 2014). The formation of this complex three-dimensional matrix creates a protective environment for pathogens to grow, which is hard to eliminate with standard antibiotics (Høiby et al., 2010). In fact, biofilm-related infections are considered a key factor in the surge of antibiotic resistance, which poses a threat to human health and to bacteria control that impacts several economic sectors, such as agriculture and food processing, whose ability to increase resistance to several antimicrobial agents has been demonstrated to be 10–1000 times higher than that of planktonic bacteria (Alves-barroco et al., 2020a). Several resistance mechanisms have been associated with biofilms, namely, by preventing the entrance of polar and charged antibiotics within the biofilm matrix or as the results of the metabolic change of bacterial growth, making these persistent infections hard to treat and eradicate (Baldassarri et al., 2006; Chadha, 2014; Rafii, 2015; Wu et al., 2015; Boonyayatra and Pata, 2016; Hwang-Soo et al., 2016; Singh et al., 2017; Alves-barroco et al., 2020b). Economic projections estimate that antibiotic resistance could cause circa 10 million deaths worldwide each year by 2050 (overshadowing rates caused by cancer-related deaths), with associated impact to healthcare systems and reduced economic growth (De Kraker et al., 2016; O’Neill, 2016). A good example of this global impact is bovine mastitis, a multifactorial disease that result from a persistent bacterial infection of the mammary glands that leads to an inflammatory response causing visibly abnormal milk (e.g., color, fibrin clots), triggering economic losses of $1.1 billion in India, $2 billion in United States and $371 million in United Kingdom (Zadoks and Fitzpatrick, 2009; De Vliegher et al., 2012; Kurjogi and Kaliwal, 2014; Gomes et al., 2016; Mushtaq et al., 2018). What is more, mastitis poses a tremendous threat to human health since it may be transmissible to humans and, thus, accountable for zoonoses and food intoxication (McDaniel et al., 2014; Gomes et al., 2016). In recent years, cases of zoonotic infections associated with SDSD have been reported, rendering it as one of the most important pathogens causing bovine mastitis (Koh et al., 2009; Zadoks et al., 2011; Park et al., 2012; Jordal et al., 2015; Chennapragada et al., 2018; Alves-barroco et al., 2021). Besides that, multidrug resistant SDSD strains with differential sensitivity to antibiotics (e.g., penicillin, oxacillin, clindamycin, vancomycin, third-generation cephalosporin) were found in human infections, only succumbing to complex antibiotic combinations (Koh et al., 2009; Park et al., 2012; Jordal et al., 2015; Chennapragada et al., 2018).

The biofilm production by SDSD strains constitute a key factor in pathogenesis, such as the capability to adhere and internalize into human and bovine cells and infect other animal hosts (Calvinho et al., 1998; Alves-barroco et al., 2018, 2019). In addition to forming a barrier against antibiotics, the biofilm also favors horizontal gene transfer, delivering compatible conditions for the uptake of genes such as high cell density, induction of competence cell, and accumulation of exogenous (Savage et al., 2013; Fair and Tor, 2014; Marks et al., 2014; Kragh et al., 2016). Thus, the use excessive of antimicrobials favors the persistence of these antibiotic resistance genes and the emergence of multidrug resistance in populations of the same ecological niches (Alves-barroco et al., 2020a). To overcome these drug resistant bacterial biofilm communities’ alternative strategies have been studied, such as the use of nanoparticles of different materials (Alves-barroco et al., 2020b). Considerable effort has focused on the development of biocidal strategies capable of mitigating biofilm formation and/or potentiating antibiotic effect against bacteria embedded in biofilms (Baptista et al., 2018). A wide range of NPs such as liposomes, metallic nanoparticles, polymeric nanoparticles, or carbon nanotubes has been proposed tackle these issues, which mostly rely on passive diffusion of nanomaterials, usually at high concentrations that pose toxicity concerns (Baptista et al., 2018; Masri et al., 2019). The antimicrobial activity of NPs against planktonic bacteria and biofilms depends on several factors, such as electrostatic attraction, van der walls forces, hydrophobic interactions, nanoparticle size, and stability (Joshi et al., 2020). The interaction between NPs and bacteria can trigger oxidative stress processes, enzymatic inhibition, cell components damage, and gene expression changes. Several NP-based systems have been applied against bacteria (for more details, see Baptista et al., 2018; Lee et al., 2019; Gómez-Núñez et al., 2020; Joshi et al., 2020). Metallic NPs (e.g., silver, silica, zinc, and gold) provide for a large surface area that increases the interaction with the bacterial membrane or matrix biofilm structures (Adeyemi and Sulaiman, 2015). Particularly, gold and silver NPs have been extensively studied for their antibacterial and anti-biofilm effects (Joshi et al., 2020). Additionally, some strategies have profited from the high extinction coefficient of these NPs and their light to heat transfer capability (Petrova et al., 2007); where the generated heat might be applied for biofilm eradication based on heat disturbance of the matrix. Indeed, gold (AuNPs) and silver nanoparticles (AgNPs) have been shown to induce biofilm disturbance upon near-infrared (Petrova et al., 2007; Adeyemi and Sulaiman, 2015; Yuwen et al., 2018; Li et al., 2019; Joshi et al., 2020). What is more, bimetallic NPs improve the original metal catalytic properties with increased stability in the surrounding media and higher activity against bacteria. Gold-silver NPs alloys (AuAgNPs) have shown good bactericide activity against bacterial biofilms (Ramasamy et al., 2016), potentiated when subjected to light irradiation (Kyaw et al., 2017), while keeping toxicity against higher eukaryotes at low impact (Taylor et al., 2015). These data suggest that using AuAgNPs to fight infections associated with biofilms would be more advantageous than the use of AuNPs and AgNPs alone. In this work, the use of AuAgNPs were investigated as the pivotal element toward eradication of bacterial biofilms. Mature SDSD biofilms were exposed to AuAgNPs alone or in combination with ciprofloxacin. The irradiation with visible light of AuAgNPs induces photothermal conversion and impart heat transfer to the biofilm, resulting in the disruption of the dense matrix, allowing the perfusion of the antibiotic, and damaging bacteria cells. Importantly, it is the combination of light stimulation that imparts the enhancement of the bactericidal efficacy of the antibiotic. Still, under these conditions, no harm is done to the mammal cells. These findings will inspire the design of next-generation synergistic strategies to tackle biofilm formation.

## Materials and Methods

### Nanoparticle Synthesis and Characterization

Gold nanoparticles (AuNPs) were synthesized according to Pedrosa et al. (2018) by boiling an aqueous solution of 98.4 mg of HAuCl_4_⋅3H_2_O (Sigma Aldrich, St. Louis, MO, United States) in 250 ml of milli-Q H_2_O and adding 38.8 mM of sodium citrate (Sigma Aldrich, St. Louis, MO, United States). The mixture was refluxed with stirring for 30 min. Silver nanoparticles (AgNPs) were synthesized according to the method described by La Spina and collaborators with several modifications (La Spina et al., 2020), by boiling an aqueous solution of 45 mg of AgNO_3_ (Merck, Darmstadt, Germany) in 250 ml of milli-Q H_2_O while stirring. Then 38.8 mM of sodium citrate was added, and the mixture refluxed for 15 min while stirring.

Gold-silver alloy NPs were synthesized following the protocol proposed by Doria et al. (2010). Briefly, 12.5 mg of HAuCl_4_⋅3H_2_O (Sigma Aldrich, St. Louis, MO, United States) and 5.4 mg of AgNO3 (Merck, Darmstadt, Germany) were dissolved in 250 ml of milli-Q H2O and brought to the boil with stirring Then 34 mM of sodium citrate (Sigma Aldrich, St. Louis, MO, United States) was added, and the mixture refluxed for 15 min with stirring.

To increase stability in complex growth media, the resulting NPs were conjugated to a thiolated polyethylene glycol (PEG-SH) (Mw 2000; 0.01 mg/mL, Sigma Aldrich, St. Louis, MO, United States). Following incubation overnight with 0.028% (w/v) Sodium dodecyl sulphate (SDS), the excess PEG was removed by centrifugation (14000 *g* for 30 min at 4°C), and the NPs washed three times with milli-Q H_2_O. The extension of surface functionalization was assessed by determination of free PEG-SH in the supernatant *via* the Ellman’s assay (Supplementary Material) as described (Moser et al., 2016).

All NPs were characterized for size and morphology by Transmission Electron Microscopy (TEM) and Dynamic Light Scattering (DLS), elemental composition by Inductively Coupled Plasma Atomic Emission Spectroscopy (ICP-AES), and optical profile by Ultraviolet-Visible Spectroscopy (UV-VIS). In all assays, the amount of metal (gold and/or silver) was kept constant (AuAgNPs, AuNPs, and AgNPs). The stability of AuAgNPs in a pH range of 2–10 was assessed by UV-VIS (Shimadzu, Kyoto, Japan) after a 48 h incubation at room temperature. For this, 10% (w/v%) HNO_3_ and 10% (w/v%) NaOH were used to adjust the target pH of a 10 nM NPs solution in ultrapure water. As control, solutions of monometallic NPs (AuNPs and AgNPs) were also prepared. Experiments were performed in triplicate and data presented as the average.

#### Transmission Electron Microscopy Characterization

Transmission electron microscopy characterization of NPs was developed in the TEM service of Instituto Superior Técnico (ICEMS/IST), Lisbon, Portugal. Hence, 10 μL of each metallic nanoparticle solution were put on carbon copper grids and washed with milli Q-water two times and air dried. Subsequently, TEM images were acquired employing a transmission electronic microscope (Hitachi, Tokyo, Japan).

#### Dynamic Light Scattering Characterization

For dynamic light scattering (DLS) analysis, a 2 nM solution of each metallic nanoparticle was prepared previously. Thereafter, the hydrodynamic diameter and Z-potential were determined by DLS resorting to a Nanoparticle Analyzer SZ-100 (Horiba Scientific, Kyoto, Japan) at 25°C with a scattering angle of 90°. Samples were measured three times.

#### Ultraviolet-Visible Spectra

The spectrum of ultraviolet-visible (UV-Vis) was measured by spectroscopy to characterize the metallic nanoparticles. Thus, 2 nM solution of each metallic nanoparticle was prepared previously diluting with milli Q-water. Afterward, UV-Visible absorption spectra were recorded at room temperature on a UV-Vis spectrophotometer (Shimadzu, Kyoto, Japan) in the range 400–800 nm with 1 cm path quartz cuvette. Three measurements of each sample were performed.

### Bacterial Strains, Growth Conditions, and Sample Preparation

The bovine SDSD isolates were selected based on the genomic profiles, antibiotic resistance profiles, and the ability to accumulate biofilms on glass and polystyrene surfaces (Alves-barroco et al., 2019, 2021; Supplementary Table 1). For all biological assays, *Staphylococcus aureus* (ATCC 29213) or *Pseudomonas aeruginosa* (ATCC 10145) [from the American Type Culture Collection (ATCC)] were used as positive biofilm producers, while an isolate of *Streptococcus pyogenes* (GAP58) was used as a stable non-biofilm producer (Alves-barroco et al., 2019). The non-biofilm isolate (GAP58) was used as a negative control.

Biofilm production was carried out as previously described with some modifications (Alves-barroco et al., 2019). Briefly, 100 μl of bacterial culture (*OD*_570_ = 0.6) was added to 100 μl of Trypticase Soy Broth–TSB (Sigma-Aldrich, St. Louis, MO, EUA) supplemented with 0.5% (w/v) glucose (Sigma-Aldrich) in a 96 well plate and mixed by pipetting. 200 μl of TSB broth supplemented with 0.5% (w/v) of glucose was used as control. The 96 well plates were sealed and incubated for 20h at 37°C. The supernatant was carefully removed, and each well was washed twice with sterile saline solution (0.85% w/v) to remove non-adherent bacteria.

### Minimum Inhibitory Concentration and Minimal Biofilm Eradication Concentration

Susceptibility tests with antibiotics were carried out in 96-well microtiter plates using a standard twofold broth microdilution method of the antibacterial agents in Mueller–Hinton (MH) broth (Difco, West Molesey KT8 2SE, United Kingdom), according to the guidelines from the Clinical and Laboratory Standards Institute (CLSI^1^). For the determination of Minimum Inhibitory Concentration (MIC) and Minimal Biofilm Eradication Concentration (MBEC) values, we used antimicrobials with bactericidal action, namely, ciprofloxacin (fluoroquinolones class) and gentamicin (aminoglycosides class), and with bacteriostatic activity, namely, tetracycline.

Serial dilutions of the antibiotics stock solutions were carried out to obtain the final concentrations of 64, 32, 16, 8, 4, 2, and 1 μg/mL. Briefly, bacterial cells were grown to the mid-exponential phase in the MH medium. Then, aliquots of 100 μl of the bacterial cells were seeded in a 96-well microtiter plate at a density of 1 × 10^6^ ml^–1^. Subsequently, 10 μl of each solution of the antibiotics prepared by serial dilutions was added to the bacterial cells. *S. aureus* ATCC 29213 was used as the positive control. The experiments were carried out in triplicates. The MICs were defined as the lowest drug concentration inhibiting visible growth after overnight incubation at 37°C. To determine the minimal biofilm eradication concentration (MBEC), the biofilms were formed as described above. After the biofilm formation, the culture medium was replaced by fresh culture medium supplemented with serial dilutions of the antibiotics stock solutions were carried out to obtain the final concentrations of 64, 32, 16, 8, 4, 2, and 1 μg/mL. The plates were incubated for 18 h at 37°C. Subsequently, the wells were washed three times with sterile PBS, then was performed the homogenization of the biofilm to disperse cells in a liquid medium and colony-forming units quantified by serial dilutions in solid medium.

### Photoirradiation

Before biofilm irradiation, actinometry was performed to measure the exact amount of energy irradiated into the system. Actinometrical measurements were performed with Aberchrome 540TM, E-form [(E)-a-(2,5-dimethyl-3-furylethylidene) (isopropylidene) succinic anhydride)] (Extrasynthese, France) actinometer as previously described (Rappon and Syvitski, 1996; Mendes et al., 2017). This photochromic dye is often used for actinometry studies in the near-UV and visible regions due to its reversible photocyclization into the deep red cyclized valence isomer 7,7a-dihydro-2,4,7,7,7a-pentamethylbenzo(b)furan-5,6-dicarboxylic anhydride (C-form). When irradiated with UV light E-form turns in C-form, which can, in turn, be reverted to E-form when irradiated with visible light. For these measurements, a solution of 100 μM of Aberchrome 540 was dissolved in absolute ethanol and irradiated at 342 nm for 1 h until a photo-stationary state corresponding to the maximum conversion into the C-form was obtained. The C-form solution was irradiated using a continuous wave (CW) 532 nm green diode-pumped solid-state laser (DPSS) (Changchun New Industries Optoelectronics Tech. Co., LTD., Changchun, China) coupled to an optical fiber. The solution exposed to the green laser undergoes back conversion to the E-form, and the number of converted molecules was quantified by UV-VIS (Shimadzu, Japan). This allows to characterize the exact amount of energy being pumped into the system by the laser, i.e., the amount of photons irradiating the NPs and cells/matrix–providing means to tune the intensity of irradiation to limit damage to healthy cells (NPs have an extinction coefficient circa 10^5 higher than any biomolecule inside cells, which allow these NPs to absorb almost all laser light and spare cells to any deleterious effect).

A thermocouple was inserted into the wells with the solution (water, culture media) before and immediately after visible light irradiation to assess the temperature increase. The thermal efficiency was calculated for the variation of initial and final time point for the pH 7 condition. The Therm(IR) camera allows assessment of temperature variation in realtime.

### Cell Viability on Primary Human Fibroblasts

Human primary fibroblasts were purchased from ATCC.^2^ The cell line was grown in Dulbecco’s modified Eagle’s medium (DMEM) (Invitrogen, Grand Island, NY, United States) supplemented with 10% (v/v) fetal bovine serum, incubated at 37°C in an atmosphere with 5% (v/v) CO_2_. Fibroblast viability was determined using 3-(4,5-dimethylthiazol-2-yl)-5-(3-carboxymethoxyphenyl)-2-(4-sulfophenyl)-2Htetrazolium, inner salt–MTS assay as previously described (Fernandes et al., 2017). Fibroblasts were seeded in 96-well plates and grown for 24 h prior to incubation for 3 h in fresh diluted medium with AuAgNPs (10 nM) or AgNPs (10 nM) [37°C in a 99% humidified atmosphere of 5% CO_2_]. Culture medium was replaced with DMEM (without phenol red) for visible light irradiation at 2.02 W cm^–2^ for 60 s. Irradiated and non-irradiated fibroblasts were incubated for another 3 h at 37°C in DMEM supplemented with 10 nM of NPs (AuAgNP, or AgNP) and 10 nM of NPs + 10 μg/mL CIP.

After the incubation period, the culture medium was removed from each well, washed with PBS, and replaced medium containing 10% MTS reagent. The 96 well plate was incubated for 60 min at 37°C in a 99% humidified atmosphere of 5% v/v) CO_2_ and the absorbance was measured directly from 96 well plates in a microplate reader at 490 nm (Infinite M200, Tecan, Switzerland). The following equation applied to calculate the cell viability (%) = 100 × (mean Abs of treatment group/mean Abs of control group, without treatment). Concentrations of AuNPs with low toxicity for animal cells tested for this study were previously tested by our research group (Pedrosa et al., 2018).

### Biofilm Irradiation

**FIGURE 1:**
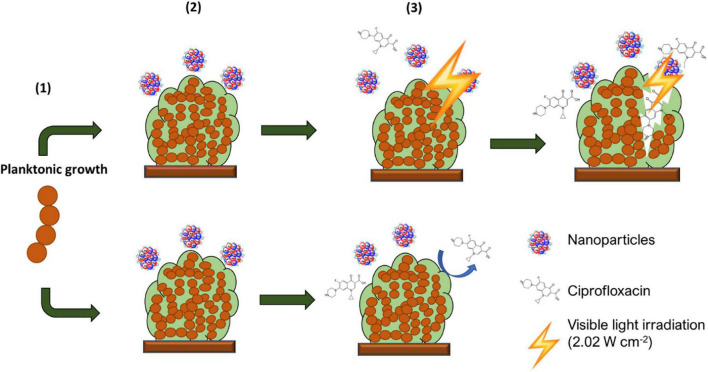
Schematic description of experimental setup. From the initial planktonic bacterial growth, biofilms were allowed to form. These were then washed to remove non-adherent bacteria and exposed to AuAgNP When indicated, these were irradiated and further cultured in presence of AuAgNPs and Ciprofloxacin (10 μg/mL).

Biofilm irradiation was carried out using a modified microdilution assay with a visible light irradiation power = 2.02 W cm^–2^ following challenging with AuAgNPs, AuNPs, or AgNPs in presence or absence of ciprofloxacin see Figure 1.

Biofilms were formed as described in the Section “Bacterial Strains, Growth Conditions, and Sample Preparation.” After 24 h of incubation at 37°C, the supernatant was carefully removed, and each well was washed twice with sterile saline solution (0.85% w/v) to remove non-adherent bacteria. Then, biofilms were incubated with fresh culture medium (TSB, pH 7.5) for 3 h at 37°C with (i) 10 nM AuAgNPs (AgNPs and AuNPs were used as controls) or (ii) TSB medium. The culture medium of biofilms was replaced with TSB only for visible light irradiation at 2.02 W cm^–2^ for 60 s. Irradiated and non-irradiated biofilms were incubated for another 3 h at 37°C in TSB supplemented with (i) 10 nM of NPs (AuAgNP, AuNP, or AgNP); (ii) ciprofloxacin (CIP, 10 μg/mL), or (iii) 10 mM of NPs + 10 μg/mL CIP. After incubation, the biofilm was detached, and cell titers were determined by serial dilution. The bioassay was performed in triplicate. The following formula calculated the percentage of biofilm reduction: Reduced cell viability (%) = 100 – {[(log_10_ CFU/mL control – log_10_ CFU/mL Treat)/log_10_ CFU/mL control] × 100}, where CFU/mL control corresponds to the number of colonies forming units per ml per milliliter of untreated biofilms, and CFU/mL Treat corresponds to the number of colonies forming units per ml per milliliter of treated biofilms.

### Inhibition of Biofilm Formation

The inhibition of biofilm formation by irradiation of AuAgNPs was evaluated on polystyrene surface following an adaptation of a previously described protocol (Genteluci et al., 2015; Alves-barroco et al., 2019). Briefly, bacterial cultures grown overnight were diluted with TSB (pH 7.5) supplemented with 0.5% (w/v) glucose (Sigma-Aldrich) to obtain *OD*_570_ = 0.6. These samples were placed in 96 well plates and incubated at 37°C for 4 h. After incubation, culture medium was replaced with TSB (pH 7.5) with 10 nM AuAgNPs without physically disrupting the newly formed biofilm. Samples were subsequently submitted to visible light irradiation (2.02 W cm^–2^) for 60 s and further incubated at 37°C for 24 h. Then, the medium was removed, samples were gently washed twice with sterile saline solution to remove the planktonic state/free-floating bacteria and dried for 30 min at room temperature. The biofilms were stained with a 0.1% solution of crystal violet for 20 min. The excess stain was removed by washing with sterile saline solution five times, and stained biofilms were dried at room temperature for one hour. Then, 200 μl of absolute ethanol was added to each stained biofilm sample. The mixture was agitated vigorously for 15 min to extract the stain. The OD of extracted crystal violet was measured at 570 nm. The following formula calculated biofilm inhibition: The percentage of biofilm inhibition = (OD Control – OD Treat)/OD Control × 100.

### Expression of *brpA*-Like and *fbpA* Genes

The biofilm formation capability of challenged samples was further assessed by monitoring the expression profile of the two main genes involved in biofilm generation—*brpA*-like and *fbpA* (Alves-barroco et al., 2019; Alves-barroco et al., 2020a). Biofilm formation was carried out for total RNA extraction and cDNA synthesis and subjected to challenge with NPs and photoirradiation as described above. Then, the medium was removed, and samples were gently washed twice with sterile saline solution to remove planktonic cells. RNA was extracted using NucleoSpin RNA II kit (Macherey-Nagel, Dueren, Germany) according to the manufacturer’s instructions. RNA was quantified in a Nanodrop Spectrophotometer (Thermo Scientific, Waltham, MA, United States). According to the manufacturer’s instructions, cDNA was synthesized from 100 ng of total RNA using SuperScript first-strand synthesis system (Invitrogen, Grand Island, NY, United States).

RT-qPCR was carried out in a total volume of 10 μL containing NZYqPCRGreen Master Mix (NZYTech, Lisbon, Portugal), 1 μL cDNA, and 0.2 μM of forward and reverse primers (Supplementary Table 2). PCR conditions included an initial denaturation at 95°C for 10 min, followed by 25 cycles of amplification consisting of denaturation at 95°C for 15 s, annealing at 55°C for 30 s, and an extension at 60°C for 45 s. The cycle threshold (Ct) was defined as the cycle in which fluorescence becomes detectable (above the background fluorescence). The expression level of each gene was normalized using the housekeeping gene *16S rRNA*. Each assay was performed with at least three independent RNA samples (and using technical duplicates).

### Sample Preparation for Transmission Electron Microscopy Analysis

Bacteria were grown in TSB supplemented with 0.5% glucose (pH 7.5) at 37°C until the mid-exponential phase was reached (*OD*_600_ of 0.5–0.6). Bacterial cells were washed three times in fresh TSB and finally resuspended TSB with AuAgNP@PEG (10 nM) or TSB alone. Then, the aliquots of cell suspension were irradiated or not with visible light (2.02 W cm^–2^) for 60 s. Irradiated and non-irradiated cells suspension were incubated for another 3 h at 37°C with TSB supplemented with AuAgNP@PEG (10 nM). Fixation, treatment of samples, and image acquisition were developed in the Electron Microscopy Facility of the Instituto Gulbenkian de Ciência (IGC), Oeiras, Portugal. Briefly, bacterial pellets were fixed for 2 h at room temperature with a solution of 2% formaldehyde, 2.5% glutaraldehyde in 0.1 M HEPES. The samples were washed in 0.1 M HEPES (2 × 5 min) and embedded in 2% low melting point agarose. Samples were post-fixed with 1% osmium tetroxide in 0.1 M HEPES for 1 h on ice, washed with 0.1 M HEPES (2 × 5 min) and dH_2_O (2 × 5 min). After washing, samples were stained with 1% Tannic Acid for 20 min on ice, washed five times with dH_2_O, and stained with 2% Uranyl Acetate for 1 h at room temperature. Samples were then dehydrated with a graded series of ethanol (30, 50, 75, 90) for 10 min each step and 100% for 10 min three times. Samples were infiltrated with 25, 50, and 75% resin in ethanol for 1 h and 30 min, and subsequently, samples were infiltrated with 100% Resin overnight. Finally, the samples were embedded with Embed-812 epoxy resin, and the blocks were polymerized at 60°C for at least 24 h. Thin sections (70 nm) were collected on slot palladium-copper grids, and post-stained with 1% uranyl acetate and Reynolds lead citrate, for 5 min each. Grids were examined in a FEI Tecnai G2 Spirit BioTWIN transmission electron microscope (120 keV) equipped with an Olympus-SIS Veleta CCD Camera.

#### Statistics

GraphPad Prism version 7.0 was used for statistical analysis. Data analyses were performed using the Student’s *t*-test, and statistical significance was considered when *p* < 0.05.

## Results and Discussion

### Characterization of AuAg-Alloy Nanoparticles

**FIGURE 2:**
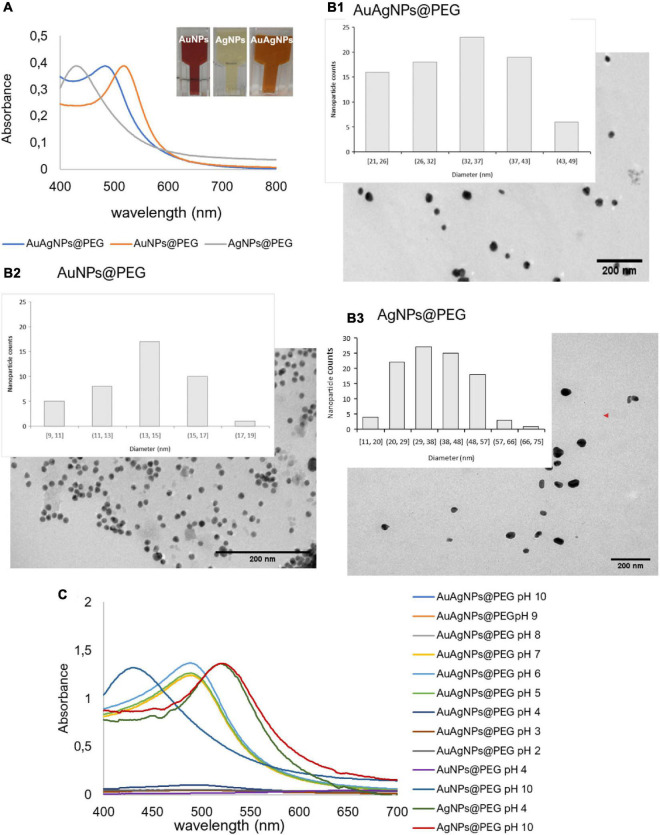
**(A)** Ultraviolet-spectrum of produced nanoparticles. AuNPs solution show an intense red color corresponding to a LSPR peak at 520 nm; AgNPs solution showed the typical faint yellow color corresponding to the LSPR peak at 430 nm; the alloy AuAgNPs resulted orange with an LSPR peak at 484 nm. **(B1–3)** Respective TEM images NPs with indication of the size distribution. **(C)** Ultraviolet spectra of NPs in different pH solutions correlating to the colloid stability of the different nanoconjugates.

A solution of spherical AuAgNPs, AuNPs, and AgNPs were prepared and conjugated with PEG-SH to achieve 100% coverage for improved stability and biocompatibility. UV-Vis spectra show the characteristic maxima absorbance peaks at 520 nm for AuNPs, 484 nm for AuAgNPs, and 430 nm for AgNPs (Figure 2A). The percentage of Au and Ag was determined by ICP-AES showing that the AuAg alloy was constituted by 51.4 ± 2.1% of gold and 48.6 ± 2.1% of silver. TEM analysis showed an average diameter of 33 (±7), 14 (±2), and 37 nm (±12) for AuAgNPs, AuNPs, and AgNPs, respectively (Figure 2B), corresponding to a hydrodynamic diameter of 19.6 (±1.8), 46.9 (±2.5), and 36.2 (±2.9) nm, respectively (Supplementary Table 3). Zeta Potential values were also determined (see Supplementary Table 3).

Considering that these nanoparticles must endure extreme salt and pH condition when diffusing into the biofilm matrices (Zhang et al., 2012), where metabolic active bacteria continuously acidify the medium (Alves-barroco et al., 2020a), the stability of the produced nanoparticles in a pH range of 2–10 was assessed through their UV-Vis profile. Despite being protected against aggregation by the PEG coverage of the surface, AuAgNPs show a tendency to aggregate at pH values lower than 4, while for pH values between 5 and 10 spectra show that the particles remain stable (Figure 2C).

The optical profile of these nanoparticles in complex media, whether aggregate or in stable colloidal dispersion, is critical for optimal photothermal conversion. In fact, irradiation at the plasmon peak ensures higher efficiency of light to heat conversion, with minimal energy dispersion due to scattering. Conversely, aggregation of nanoparticles, with concomitant red-shift of the SPR peak and augmented scattering cross-section leads to energy dissipation and, thus, lower photo-thermal conversion efficiencies (Yang et al., 2014; Guo et al., 2017). Thus, since irradiation was set at 532 nm, stability of the nanoparticles against pH induced aggregation and the effect on the heating capability was evaluated. For AuAgNPs, the photothermal effect decreased below pH 5 (Supplementary Table 4). Similarly, acidic pH promoted the aggregation of AgNPs, which lowered their photothermal effect, but at higher pH values, they were stable (Figure 2C). Contrary, AuNPs showed good stability for all pH conditions studied, which did not influence their heat capacity (Supplementary Table 4). The stability of alloy nanoparticles upon irradiation (different laser powers) was also assessed by UV-Vis spectra and DLS (Supplementary Figure 1). Data show only but minor variations to the AuAgNPs’ profiles, indicating that these nanoparticles are stable after irradiation.

### Biological Activity of Nanoparticles and Ciprofloxacin

To assess the effect of visible light irradiation of AuAgNPs on biofilm disruption, extreme care was taken to use nanoparticle concentrations that would not influence microbial growth. Despite, several reports on the antimicrobial activity of noble metal nanoparticles (Castillo-Martínez et al., 2015; Le Ouay and Stellacci, 2015; Quinteros et al., 2016; Ramasamy et al., 2016; Alhmoud et al., 2017; Wang et al., 2019), the biocidal effect strongly depends on size, functionalization, and concentration of nanomaterial. For our studies, we selected NP concentrations that did not hamper planktonic bacteria growth, thus allowing to clearly highlight the synergistic effect of photoirradiation. Indeed, the effects of NPs alone in reference bacterial strains are negligible–see Supplementary Figure 2.

Although several studies have shown high activity of NPs (AuNPs, AgNPs, and AuAgNPs) against Gram-positive and Gram-negative bacteria in planktonic or sessile growth (Joshi et al., 2020), the possible toxicological implications of the high concentrations used may hinder direct application in the clinics. Most studies tested high concentrations of metallic nanoparticles to eradicate the bacteria in biofilms (Ramasamy et al., 2016; Singh et al., 2018). Therefore, we assessed the effect of AuAgNPs, AgNPs and the effect of visible light irradiation of NPs on viability of primary human fibroblasts that, for the tested concentrations, did not show any impact to cell viability (Supplementary Figure 3). These observations are in line with data in the literature on the toxicity of noble metal nanoparticles on mammal cells (Conde et al., 2014; Tiedemann et al., 2014; Taylor et al., 2015; Alhmoud et al., 2017; Fernandes et al., 2020; Chang et al., 2021).

Similarly, we assessed the MIC of ciprofloxacin, gentamicin, and tetracycline of bovine SDSD isolates, *S. aureus* (ATCC 29213), and *P. aeruginosa* (ATCC 10145) (Table 1). According to the Clinical and Laboratory Standards Institute (CLSI M31-A3, 2008) criteria, resistance values for interpretation of MIC results were: ≥4 for ciprofloxacin (CIP), ≥16 for gentamicin, and ≥8 for tetracycline. Tetracycline resistance was found in all SDSD isolates/strains. High-level gentamicin resistance was observed for VSD22 and VSD45 (>16), and a high-level erythromycin resistance was shown for VSD9 and VSD13 (>16). According to the CLSI standard, all the studied isolates/strains were ciprofloxacin-susceptible (Table 1).

**TABLE 1:** Minimum inhibitory concentration values for ciprofloxacin, gentamicin, and tetracycline.

	*MIC values (μg/mL)*		
	
*Strains*	Ciprofloxacin	Gentamicin	Tetracycline
*SDSD VSD9*	<1	2	32
*SDSD VSD13*	<1	2	64
*SDSD VSD16*	<1	2	64
*SDSD VSD22*	<1	>16	32
*SDSD VSD45*	<1	>16	64
*S. aureus ATCC 29213*	<1	2	4
*P. aeruginosa ATCC 10145*	<1	2	4

***Resistant:** Ciprofloxacin ≥ 4; Gentamicin ≥ 16; Tetracycline ≥ 8.*

The minimal concentration of antibiotics required to eradication of biofilm cell (MBEC) was also assessed. MBEC is the lowest concentration of an antimicrobial that inhibits visible after to collect biofilm cells (Alves-barroco et al., 2020a). Our results reveal that, under these criteria, MBEC was not achieved for the tested antibiotics, even at the highest concentrations tested.

The susceptibility of SDSD strains to different antibiotics by disk diffusion method was previously investigated (Alves-barroco et al., 2021). Herein, we used antibiotics whose resistance has been associated with the formation of biofilms, such as ciprofloxacin that shows similar bactericidal action on planktonic growths of isolates/strains and loss of bacterial cell activity in biofilms.

### Photoirradiation Effects of AuAgNPs in Mature Biofilms

**FIGURE 3:**
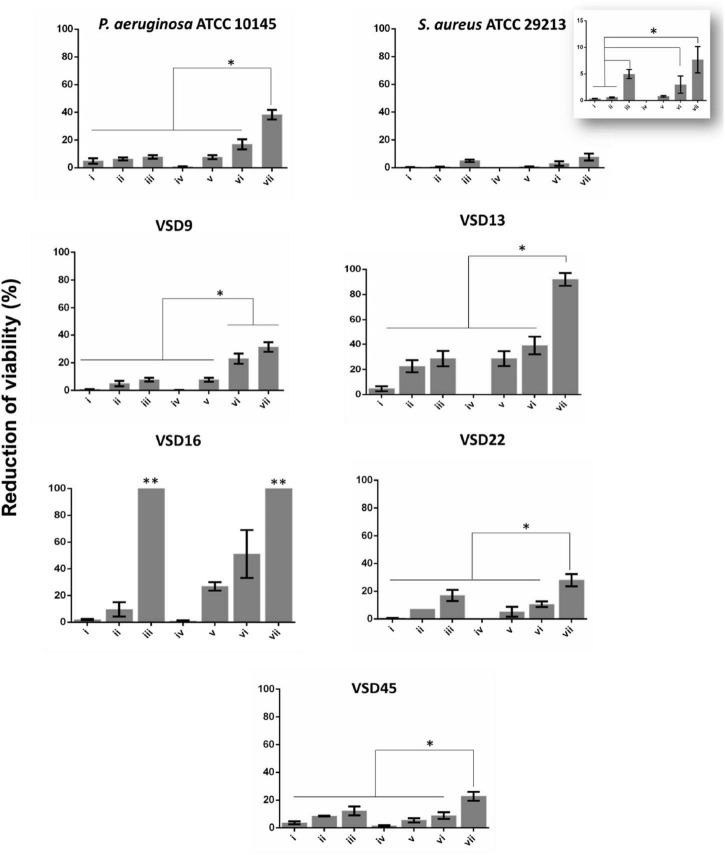
Biofilm destruction (%). (i) AuAgNPs; (ii) ciprofloxacin (CIP); (iii) AuAgNPs + CIP; (iv) visible light irradiation (VLI); (v) AuAgNPs + VLI; (vi) VLI + CIP; (vii) AuAgNPs + VLI + CIP. Nanoparticles were at 10 nM and VLI at 2.02 W cm^–2^. Data represented as the mean ± standard deviation (SD) of three independent measurements. The following formula calculated the percentage of biofilm reduction: Reduced cell viability (%) = 100–{[(log_10_ CFU/mL control – log_10_ CFU/mL Treat)/log_10_ CFU/mL control] × 100}, where CFU/mL control corresponds to the number of colonies forming units per ml per milliliter of untreated biofilms, and CFU/mL Treat corresponds to the number of colonies forming units per ml per milliliter of treated biofilms. *p < 0.05, Significant differences regarding each condition as a single strategy or when compared to treatment with two conditions simultaneously. ^**^p < 0.05 Significant differences AuAgNPs + CIP or AuAgNPs + VLI + CIP when compared to treatment with other conditions.

The *in vitro* therapeutic potential of photoirradiation in the visible (2.02 W cm^–2^ for 60 s) of NPs (AgNPs, AuNPs, and AuAgNPs) alone or in combination with ciprofloxacin was evaluated in mature biofilms of *S. aureus* ATCC 29213 and *P. aeruginosa* ATCC 10145 (Figure 3 and Supplementary Figure 2). No statistically significant differences were observed for AuAgNPs, AgNPs, and AuNPs when used as a single strategy or in combination with ciprofloxacin and/or photoirradiation in the eradication of mature biofilms from *S. aureus* ATCC 29213 and *P. aeruginosa* ATCC 10145 (Figure 3 and Supplementary Figure 2).

Data show that AuAgNPs alone have little to no effect on SDSD biofilm stability and bacteria viability (Figure 3–condition i). Results show a reduction of viability of mature biofilms produced by SDSD (less than 5% reduction) upon exposure to AuAgNPs similar to that of control strains (*S. aureus* ATCC 29213 and *P. aeruginosa* ATCC 10145). Interestingly, CIP (Figure 3 condition ii) showed different degrees of antibacterial activity for SDSD isolates with 5% reduction of VSD9 viability to 22.5% reduction in VSD13, but still higher than values for *S. aureus* ATCC 29213 (0.57%) and for *P. aeruginosa* ATCC 1014 (4.95%). Combinations of AuAgNPs and CIP (Figure 3 condition iii) leads to higher inhibitory activity than AuAgNPs alone (Figure 3 condition i). Data for control strains also show that no relevant effect is observed for CIP alone or in combination with AuAgNP.

Interestingly, the higher synergistic interaction was observed against VSD16 (100%), which exhibits a matrix richer in extracellular DNA (Alves-barroco et al., 2019). It is known that regardless of the bacterial species, all biofilms share common properties; thus, biofilms interact specifically (e.g., ligand-receptor) or not specifically (e.g., electrostatic interactions) with biotic and abiotic surfaces. However, the composition of biofilm matrices can vary dramatically from strain to strain.

We have previously shown that the extracellular matrix of SDSD biofilms is rich in proteins and a mucus-like extracellular component associated to extracellular DNA (eDNA), whose proportion in VSD16 biofilm showed a massive amount of the latter (Alves-barroco et al., 2019). AuNPs and AgNPs coated by molecules with positive electrostatic charges play the primary role in the interaction to eDNA with a polyanionic nature. The eDNA also interacts with NPs *via* Van der Waals forces or hydrophobic forces, preventing the aggregation of NPs in the solution. Some authors attributed the biofilm inhibition to the interactions of Au^+^ and Ag^+^ with eDNA, and these interactions are believed to play a predominant role in inhibiting eDNA function (Jiang and Ran, 2018; Radzig et al., 2019).

Silver and gold are well-known to promote bactericidal activity against Gram-negative and Gram-positive, although the mechanisms are not yet fully elucidated. Some authors have suggested that the bactericidal activity is mediated by the production of reactive oxide species (ROS) like superoxide or hydrogen peroxide by silver ions that interact and inactivate some cellular components (e.g., proteins, lipids, and nucleic acids) and affect the electron transport chain causing cell death (Costa et al., 2010; Le Ouay and Stellacci, 2015; Quinteros et al., 2016). Another hypothesis relates to the fact that increasing levels of oxygen decrease the bacteria’s resistance to different antibiotics classes, since some antibiotics, such as ciprofloxacin (and aminoglycosides in general) are not active in anaerobic conditions, thus affecting only the outer part of the biofilm (Borriello et al., 2004). To our knowledge, this study reports for the first-time ciprofloxacin resistance associated with biofilm formation by SDSD strains. Taken together, our data suggest that AuAgNPs could interact with the biofilm matrix increasing the permeability and diffusion of the antibiotic into the biofilm, thus capable to kill the bacteria, since the VSD16 strain is sensitive to ciprofloxacin in its planktonic form.

Our data show that photoirradiation of AuAgNPs in combination with ciprofloxacin leads to extensive biofilm degradation and loss of bacterial cells’ viability, reaching eradication values of 100% for some strains–Figure 3 condition vii. No statistical differences are observed in the number of viable cells after irradiation compared to non-irradiated biofilms (Figure 3–condition iv).

We focused on light irradiation combination with metal nanoparticles since no mechanism of resistance has yet been identified. The photothermal conversion mediated by metal nanoparticles (Kim et al., 2019; Yang et al., 2020) triggers a local surge in temperature (Supplementary Table 4) that impacts bacteria cells’ viability and the stability of the extracellular matrix, decreasing its thickness (Jo and Kim, 2013; Wang et al., 2019; Beckwith et al., 2020; Pinel et al., 2021). Previous studies have shown that the structure and the integrity of bacterial biofilms could be disrupted at higher temperatures (Ibelli et al., 2018), thus decreasing the barrier limiting antimicrobial diffusion to different layers of the biofilm. In fact, some studies have demonstrated the possibility to use near-infrared irradiation to induce nanoparticle heat transfer (Castillo-Martínez et al., 2015; Alhmoud et al., 2017; Ding et al., 2017; Astuti et al., 2019; Li et al., 2019). Our data suggest that the nanomaterial could interact with the biofilm matrix increasing the permeability and diffusion of the antibiotic into the biofilm, then becoming capable to kill the bacteria, since the VSD16 strain is sensitive to CIP in its planktonic form. Previous studies reveal that in immature biofilms, the number and dimensions of pores are larger, which facilitates diffusion of nanoparticles (Peule and Wilkinson, 2011). Also, NPs deposition inside the biofilms depends on the biofilm surface’s electrostatic interaction, so does adhesion of bacteria, and, thus, metal NPs might contribute to a change to the environment inhibiting bacterial adhesion and consequently the formation of new biofilms.

Here, we use visible light irradiation (2.02 W cm^–2^ for 60 s) of the AuAgNPs (10 nM) to induce antibiofilm activity against *S. aureus* ATCC 29213, *P. aeruginosa* ATCC 10145, and SDSD strains.

### Effect of Photoirradiation of AuAgNPs on *de novo* Biofilm Formation

**FIGURE 4:**
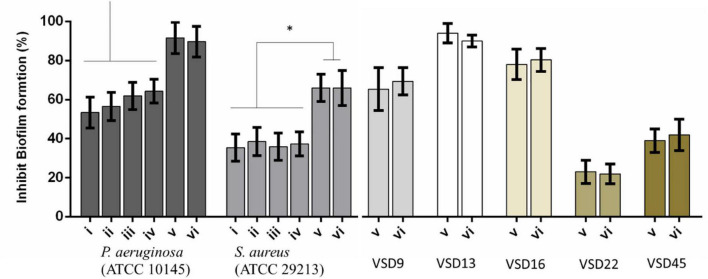
Percent inhibition of biofilm formation of (i) AuNPs; (ii) AuNPs + visible light irradiation (VLI); (iii) AgNPs; (iv) AgNPs + VLI; (v) alloy AuAgNPs; (vi) alloy AuAgNPs + VLI. To assess biofilm formation, the absorbance was measured at 570 nm. All nanoparticles were at 10 nM and VLI at 2.02 W cm^–2^. *p < 0.05.

One critical aspect when dealing with infectious biofilms is their capability to be formed, even upon disinfection and cleaning of surfaces and tissues. So, the effect of photoirradiation of AuAgNPs on *de novo* biofilm formation was assessed–Figure 4.

Treatment with AuAgNPs immediately upon seeding on surfaces showed to be an obstacle (inhibition of 89–91%) for biofilm growth and development. What is more, the alloy nanoparticles showed to have a statistically more pronounced effect against biofilm formation when compared to the single metal counterparts–Figure 4. More significant biofilm formation inhibition was observed for VSD13 (weak biofilm producer) and VSD16 (moderate biofilm producer) than for VSD22 (strong biofilm producer) (see also Supplementary Table 1). Together, these results seem to indicate that challenge with AuAg-alloy NPs yield less robust biofilm.

Interestingly, there was no observable difference for irradiated vs. non-irradiated samples. This observation corroborates the idea that photoconversion within the biofilm is critical for the matrix stability and support. When irradiation occurs prior to biofilm establishment, since no organized extracellular matrix is present, the effect is solely attributed to the impact of the nanomaterial on bacterial cells’ viability (Yuwen et al., 2018; Kirui et al., 2019; Ortiz-Benítez et al., 2019).

**FIGURE 5:**
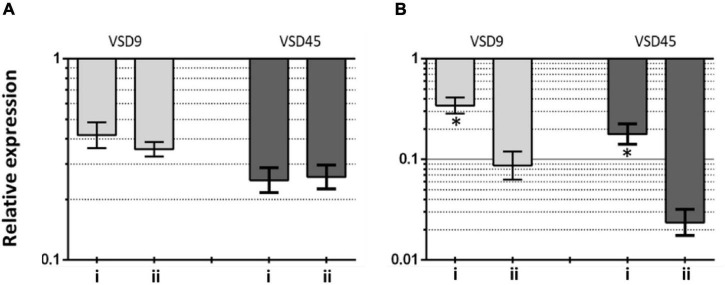
Transcriptional profiling of **(A)** *brpA*-like (encoding biofilm regulatory protein) and **(B)** *fbpA* (fibronectin-binding protein A) in the SDSD strains AuAgNPs combined with visible light irradiation (VLI) relative to the untreated. 16S rRNA gene was used as the internal control for normalization. Relative expression was calculated by the 2^–ΔΔCt^ method. (i) AuAgNP and (ii) AuAgNP and VLI. Nanoparticles were at 10 nM and VLI at 2.02 W cm^–2^.

This hypothesis seems to be further supported on data from gene expression analysis on the effects of AuAgNPs upon critical genes involved in biofilm formation, *brpA*-like, which encodes a biofilm regulatory protein that is an important regulator of biofilm formation, and *fbpA*, a fibronectin-binding protein A pivotal for the adherence process. The expression of these genes was evaluated in VSD9 and VSD45 challenged with AuAgNPs with and without photoirradiation–Figure 5. Challenging bacteria with AuAgNPs triggered downregulation of both genes, which is more pronounced for non-irradiated samples *brpA-like* downregulated ∼0.45 and 0.32-fold for AuAgNPs alone and AuAgNPs + irradiation, respectively; *fbpA* gene downregulated ∼0.2–0.4-fold for AuAgNPs alone and ∼0.025–0.075-fold for AuAgNPs+ irradiation. These data are in line with the observed decrease in biofilm formation that show that the alloy nanoparticles have a more pronounced effect in decreasing the capability of bacteria cells to produce viable biofilms, probably mediated by impacting gene expression. These results agree with previous studies that report that NPs can modulate bacterial properties since the adhesion to different surfaces requires regulated events in response through crucial regulatory genes (Singh et al., 2018). Data indicate that SDSD biofilm reduction by AuAgNPs may trigger distinct stress pathways that lead to downregulation of these two genes critical for biofilm production, resulting in less robust biofilm. Still, further studies are required to assess the true potential of AuAgNPs in the regulation of genes associated with the formation of biofilms.

### Transmission Electron Microscopy Microphotographs of *Streptococcus dysgalactiae* subsp. *dysgalactiae* Exposed to AuAgNPs and Photoirradiation

**FIGURE 6:**
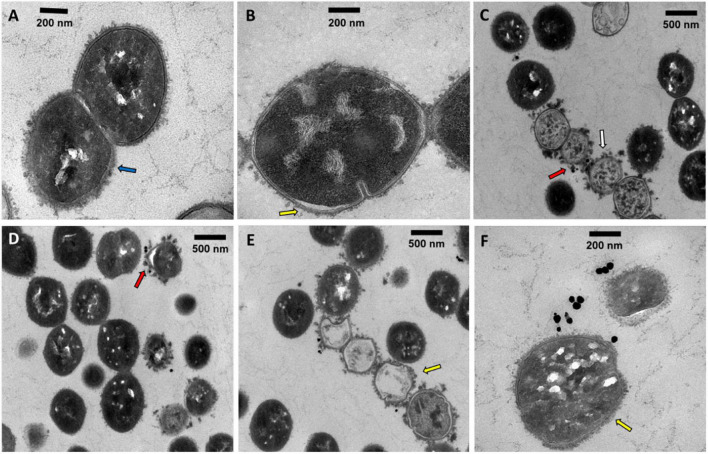
Transmission electron microscopy microphotographs of SDSD VSD22 cells **(A)** untreated; **(B,C)** treated with AuAgNPs (10 nM) combined with visible light irradiation at 2.02 W cm^–2^ for 60 s; **(D–F)** treated with AuAgNPs alone. Blue arrow shows glycocalyx from untreated cells; Yellow arrow shows cell envelope not defined with wrinkling of the cell wall; Red arrows show nanoparticles adhered to the cell envelope; White arrow shows aggregation of the cytoplasmic components.

To assess the localization and eventual interaction between NPs and bacteria, SDSD VSD22 cells treated with AuAgNPs with and without photoirradiation were examined by TEM. Overall, TEM images show that the AuAgNPs were not internalized by the SDSD VSD22 cells but seem to be adhering to the outer structures and components, such as cell envelope. This is agreement with previous studies that show that nanoparticles of 30 nm in diameter and larger do not penetrate bacteria cells due to the dimensions of pores within the bacterial cell wall (sized between 4 and 16 nm) (Turner et al., 2013; Pajerski et al., 2019). Also, it has been demonstrated that noble metal NPs interact with various proteins outside the bacteria cells which might modify their structural features, thus hampering the function in biofilms assembly (Joshi et al., 2020). Even though some alteration to the structure of the SDSD VSD22 cells after treatment were observed (Figure 6), these did not affect the growth of this strain. Untreated cells exhibited a uniform cytoplasm, while aggregation of the cytoplasmic components was observed among treated cells (see Figure 6C; España-Sánchez et al., 2017). It has been suggested that these cytoplasmic alterations might be associated to some degree of damage to the cell membrane (Dakal et al., 2016; Wang et al., 2016).

Transmission electron microscopy microphotographs show that exposure to AuAgNPs followed by photoirradiation triggered some alterations to the cell walls. The SDSD VSD22 untreated cells exhibit a well-defined wall with a continuous cell envelope (Figure 6A), whereas challenged cells exhibit less defined cell envelope with wrinkling of the cell wall (Figures 6D,E). This alteration could be partially justified by the decreased expression of the *brpA* gene since BrpA proteins have an important role in cell-wall biogenesis and structure (Bitoun et al., 2012). The observed structural features also support the observed decrease in biofilm formation, suggesting an important role in the ability of bacterial biofilm production.

## Conclusion

In this study, we evaluated the impact of AuAg-alloy nanoparticles in the biofilm forming capability of bacterial strains that could be potentiated by light irradiation. Under the tested condition (10 nM), these AuAg-alloy nanoparticles had only but negligible effect on the planktonic growth of the bacterial strains. However, an increase in the reduction to the number of viable cells of *S. aureus* ATCC 29213 and *P. aeruginosa* ATCC 10145 was observed when biofilms were treated with gold:silver alloy nanoparticles (10 nM) + ciprofloxacin (10 μg/mL) + visible light irradiation (2.02 W cm^–2^). Similar results were obtained for SDSD biofilms when treated with AuAgNPs combined with the antibiotic and irradiated. Notably, the higher synergistic interaction was observed against VSD16 (100%), which exhibits a matrix richer in extracellular DNA, which may be more sensitive to thermo-denaturation and, thus, disruption of its structural features. We also evaluated the effects of AuAgNPs alone or with irradiation on the expression of two genes involved in biofilm formation by SDSD strains, *brpA*-like and *fbpA* fibronectin-binding protein A, revealing that both genes were downregulated upon insult resulting in less robust biofilm.

Our findings suggest that a combination of AuAg-alloy NPs and photoirradiation provides disturbance of the biofilms’ matrix, increasing the permeability and entry of antibiotic into the biofilm. Besides, we demonstrated that AuAgNPs were also found to be effective for biofilms inhibition. Our results suggest that AuAgNPs activity downregulate genes critical for SDSD biofilm production, namely, *brpA*-like encoding biofilm regulatory protein and *fbpA* fibronectin-binding protein A.

As such, this study provides useful information to assist the development of nanoparticle-based strategies for the active treatment of biofilm-related infections triggered by photoirradiation in the visible. Because visible light is used, such application could include the direct visualization and removal of biofilms (e.g., udders, surface sterilization, etc.).

## Data Availability Statement

The original contributions presented in the study are included in the article/Supplementary Material, further inquiries can be directed to the corresponding authors.

## Author Contributions

CA-B contributed to biological assays with human fibroblasts cells and bacteria under the supervision of AF and PB, and contributed to drafting the manuscript. LR-G contributed to nanoparticle synthesis and characterization. AF and PB revised the literature and drafted and edited the final manuscript. All authors contributed to the article and approved the submitted version.

## Conflict of Interest

The authors declare that the research was conducted in the absence of any commercial or financial relationships that could be construed as a potential conflict of interest.

## Publisher’s Note

All claims expressed in this article are solely those of the authors and do not necessarily represent those of their affiliated organizations, or those of the publisher, the editors and the reviewers. Any product that may be evaluated in this article, or claim that may be made by its manufacturer, is not guaranteed or endorsed by the publisher.

## Footnotes

1
http://www.clsi.org/

2
http://www.atcc.org
