# Supplementary material for: Light Triggered Enhancement of Antibiotic Efficacy in Biofilm Elimination Mediated by Gold-Silver Alloy Nanoparticles
Source: Front Microbiol. 2022 Feb 28;13:841124. doi: 10.3389/fmicb.2022.841124 (PMC8919054; doi:10.3389/fmicb.2022.841124)
Supplement: Supplementary file 1 [file Data_Sheet_1.docx]

**Supporting Information**

**Light triggered enhancement of antibiotic efficacy in biofilm elimination mediated by gold-silver alloy nanoparticles.**

Cinthia Alves-Barroco^1,2^, Lorenzo Rivas-García^1,3^, Alexandra R. Fernandes^1,2*^, Pedro Viana Baptista^1,2*^

^1^UCIBIO, Dept. Ciências da Vida, Faculdade de Ciências e Tecnologia, Universidade NOVA de Lisboa, 2819-516 Caparica, Portugal

^2^i4HB, Associate Laboratory - Institute for Health and Bioeconomy, Faculdade de Ciências e Tecnologia, Universidade NOVA de Lisboa, 2819-516 Caparica, Portugal

^3^Biomedical Research Centre, Institute of Nutrition and Food Technology, Department of Physiology, Faculty of Pharmacy, University of Granada, Avda. del Conocimiento s/n. 18071 Armilla, Granada, Spain.

*Co-last and corresponding authors - E-mail: [ma.fernandes@fct.unl.pt](mailto:ma.fernandes@fct.unl.pt) (ARF); [pmvb@fct.unl.pt](mailto:pmvb@fct.unl.pt) (PVB)

**Table S1.** Isolates selected for the analysis of the eradication of the biofilms formed, with information on the PFGE profiles, antibiotic resistance profiles, and the ability to accumulate biofilms in vitro.

|  |  |  |  | Biofilm formation | | | | | |
| --- | --- | --- | --- | --- | --- | --- | --- | --- | --- |
|  |  |  |  | Glass surface | | | Polystyrene surface | | |
| Strain | Year of isolation | PFGE type | Resistance phenotype | Phenotype | OD600 ± SD | | Phenotype | OD570± SD | |
| VSD9 | 2002 | N1 | cMLSB + tet | Strong | 0,75 | 0,03 | Strong | 0,34 | 0,08 |
| VSD13 | 2002 | I1 | cMLSB + tet | Weak | 0,22 | 0,05 | Weak | 0,08 | 0,04 |
| VSD16 | 2002 | A2 | tet | Strong | 0,88 | 0,08 | Moderate | 0,12 | 0,03 |
| VSD22 | 2013 | Q1 | tet + gen | Strong | 0,92 | 0,09 | strong | 1,06 | 0,65 |
| VSD45 | 2012 | AH1 | tet + gen | Weak | 0,14 | 0,02 | Moderate | 0,17 | 0,05 |

Interpretation of biofilm formation on polystyrene was performed according to the criteria previously described, and the isolates were therefore categorized as follows: non-producer: OD ≤ OD_ctrl_, (all strains which OD values were below 0.060); weak producer: OD_ctrl_ < OD ≤ 2 × OD_ctrl_, (all strains which OD values were above 0.060 and below 0.120); moderate producer: 2 × OD_ctrl_ < OD ≤ 4 × OD_ctrl_ (all strains which OD values were above 0.120 and below 0.240), strong producer: OD > 4 × OD_ctrl_ (all strains which OD values were above 0.240) and interpretation of biofilm formation on glass were performed according to the criteria: OD_600_≤0.099, no formation; OD_600_ between 0.1–0.299, weak; OD_600_ between 0.3–0.599, moderate; OD_600_>0.600, strong. cMLSB: resistance constitutive to macrolide, lincosamide, and streptogramin B. tet: resistance to tetracycline. gen: resistance to gentamicin. VSD9, VSD13, VSD16 were partially characterized in Rato et al., 2013; Alves-Barroco et al., 2019; Alves-Barroco et al., 2021.

**Table S2.** Primer sequences used for RT-PCR analysis in this study

| **Primer name** | **Sequence (5’-3’)** | **PCR product size (bp)** |
| --- | --- | --- |
| fbpA-like |  |  |
| for | CGCACCATTTTACCAGGCTC | 376 |
| rev | TCAAGTCACTCGCTTGCTGA |  |
| brpA-like |  |  |
| for | TGAAGCTAAGTTGAATGCTGC | 534 |
| rev | GAACCACCATCAGACAAGGT |  |

*brpA*-like - encoding biofilm regulatory protein

*fbpA*-like - fibronectin-binding protein A

**Table S3.** Characterization of gold, silver and alloy nanoparticles by Dynamic Light Scattering and Zeta Potential

|  | **DLS** | | **Zeta** | |
| --- | --- | --- | --- | --- |
|  | Average (nm) | St Dev | Average (mV) | St Dev |
| AuNPs | 16.8 | 1.7 | -57.2 | 2.1 |
| AuNPs@PEG | 19.6 | 1.8 | -79.0 | 2.7 |
| AgNPs | 24.1 | 2.7 | -53.5 | 3.4 |
| AgNPs@PEG | 36.2 | 2.9 | -65.5 | 2.6 |
| AuAgNPs | 36.9 | 3.0 | -21.8 | 2.6 |
| AuAgNPs@PEG | 46.9 | 2.5 | -47.2 | 2.7 |

**Table S4.** Heat capacity of AuAgNPs@PEG and AuNPs@PEG produced by irradiation at 532 nm (respect pH 7 solution). Heat capacity of AuAgNPs@PEG decreased for upon pH 4. Heat capacity of AuNPs@PEG remained unalterable at all pH situation studied.

|  | **Reduction of heat induction* (% respect pH 7 solution)** | |
| --- | --- | --- |
| **pH** | **AuAgNPs@PEG** | **AuNPs@PEG** |
| 10 | 88 | 97 |
| 9 | 87 |  |
| 8 | 88 |  |
| 6 | 85 |  |
| 5 | 61 |  |
| 4 | 20 | 99 |
| 3 | 16 | 98 |
| 2 | 18  *$Reduction heat induction\left( \% \right)=\frac{\Delta tempeture pH X}{\Delta temperature pH 7}x100$ | 98 |


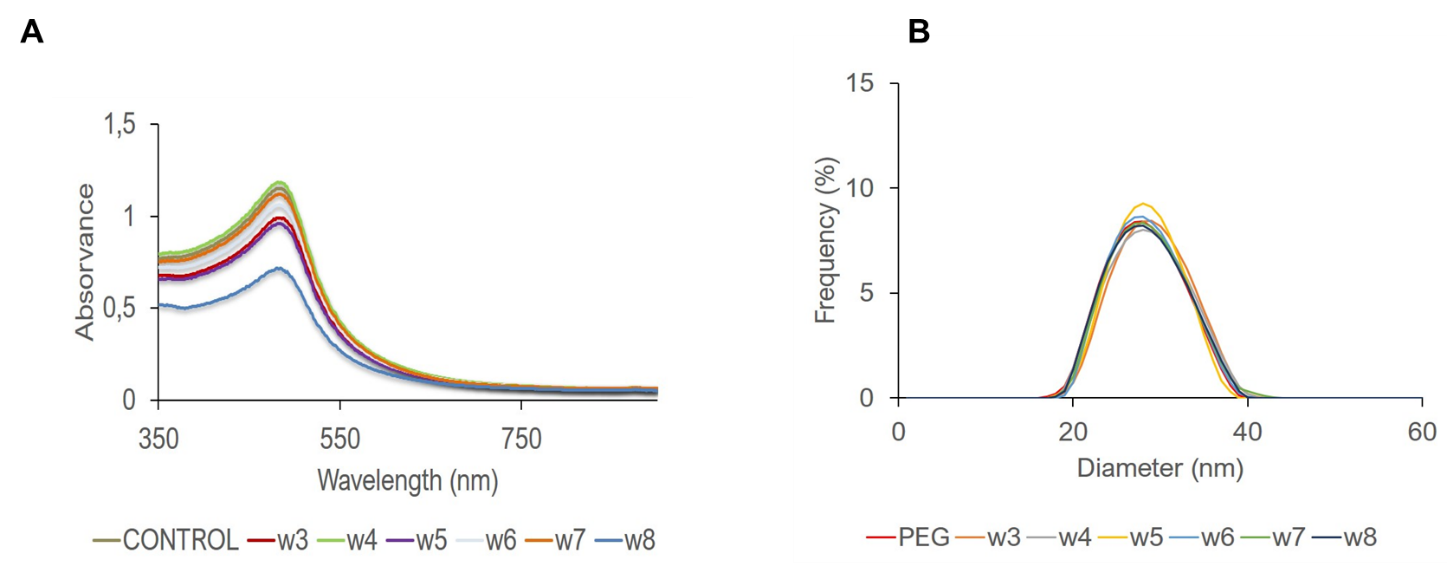


**Figure S1. A)** Visible spectrum of AuAgNPs@PEG after irradiation using different laser powers (w4-8) **B)** Size distribution determined by DLS of AuAgNPs@PEG after irradiation using different laser powers (w4-w8).


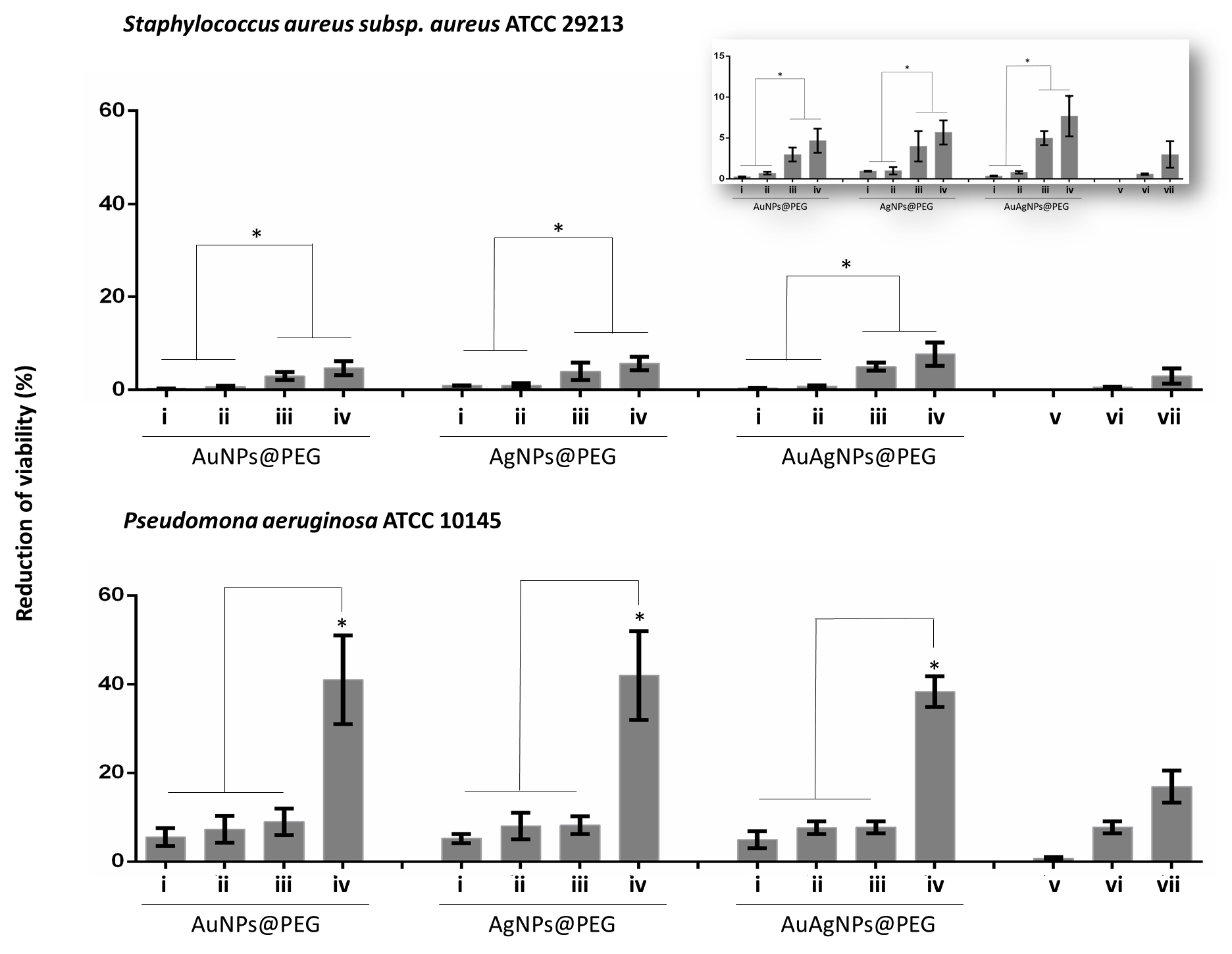


**Figure S2. Biofilm destruction** **(%)**: **(i)** NPs@PEG: Nanoparticles; **(ii)** NPs@PEG + visible light irradiation (VLI); **(iii)** NPs@PEG + ciprofloxacin (CIP)**; (iv)** NPs@PEG **+** VLI **+** CIP; **(v)** VLI; **(vi)** CIP**; (vii)** VLI + CIP**.**  Nanoparticles were at 10 nM, ciprofloxacin at 10 μg/mL and VLI at 2.02 W cm^-2^. Data represented as the mean ± standard deviation (SD) of three independent measurements. The following formula calculated the percentage of biofilm reduction: Reduced cell viability (%) = 100 – [((log_10_ CFU/mL control – log_10_ CFU/mL Treat) / log_10_ CFU/mL control) × 100], where CFU/mL control corresponds to the number of colonies forming units per ml per milliliter of untreated biofilms, and CFU/mL Treat corresponds to the number of colonies forming units per ml per milliliter of treated biofilms. **p* < .05, Significant differences.


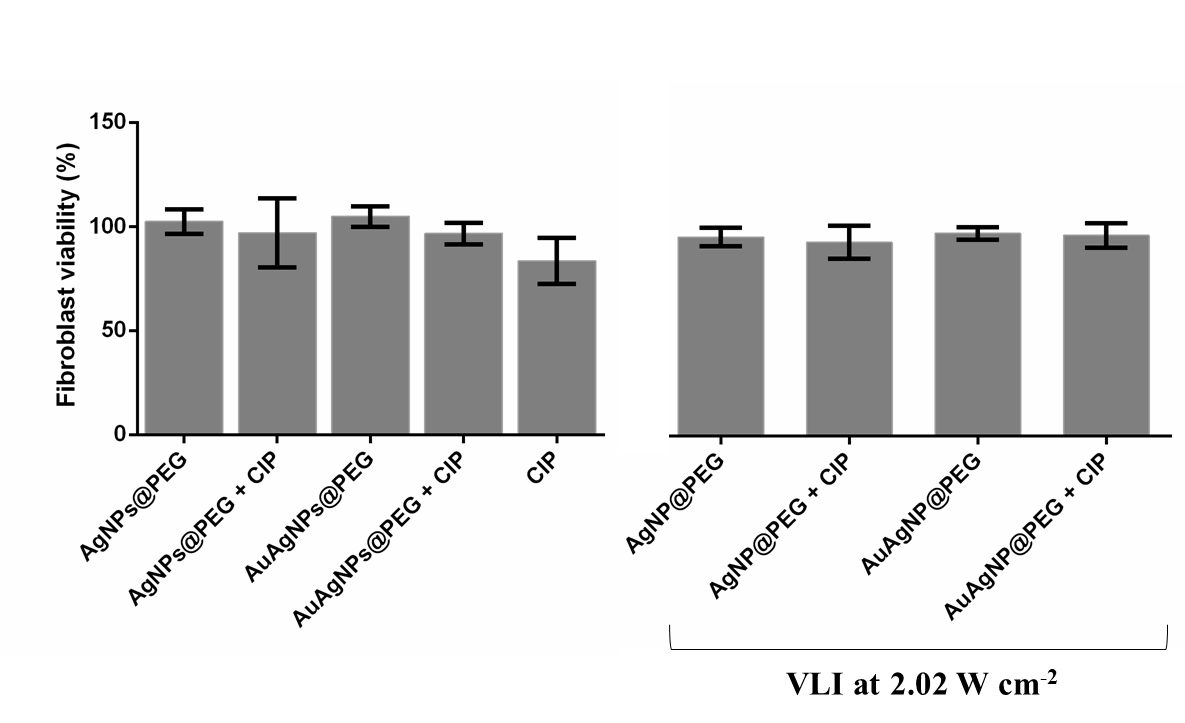


**Figure S3.** MTS assay of human primary fibroblasts previous 6h exposure to AuAg@PEG (10 nM), Ag@PEG (10 nM), CIP - ciprofloxacin (10 μg/mL), AuAg@PEG + CIP or Ag@PEG + CIP. VLI - visible light irradiation at 2.02 W cm^-2^. The following equation applied to calculate the cell viability (%) = 100 x (mean Abs of treatment group /mean Abs of control group, without treatment). Data are the average of at least three independent assays and error bars correspondent to standard deviation.
